# Addressing the Selectivity of Enzyme Biosensors: Solutions and Perspectives

**DOI:** 10.3390/s21093038

**Published:** 2021-04-26

**Authors:** Bogdan Bucur, Cristina Purcarea, Silvana Andreescu, Alina Vasilescu

**Affiliations:** 1 National Institute for Research and Development in Biological Sciences, 296 Splaiul Independentei, 060031 Bucharest, Romania; bucurica@yahoo.com; 2 Institute of Biology, 296 Splaiul Independentei, 060031 Bucharest, Romania; cristina.purcarea@ibiol.ro; 3 Department of Chemistry and Biomolecular Science, Clarkson University, Potsdam, NY 13676, USA; eandrees@clarkson.edu; 4 International Centre of Biodynamics, 1B Intrarea Portocalelor, 060101 Bucharest, Romania

**Keywords:** selectivity, enzyme, electrochemical biosensor, enzymatic inhibition, biocatalytic sensor

## Abstract

Enzymatic biosensors enjoy commercial success and are the subject of continued research efforts to widen their range of practical application. For these biosensors to reach their full potential, their selectivity challenges need to be addressed by comprehensive, solid approaches. This review discusses the status of enzymatic biosensors in achieving accurate and selective measurements via direct biocatalytic and inhibition-based detection, with a focus on electrochemical enzyme biosensors. Examples of practical solutions for tackling the activity and selectivity problems and preventing interferences from co-existing electroactive compounds in the samples are provided such as the use of permselective membranes, sentinel sensors and coupled multi-enzyme systems. The effect of activators, inhibitors or enzymatic substrates are also addressed by coupled enzymatic reactions and multi-sensor arrays combined with data interpretation via chemometrics. In addition to these more traditional approaches, the review discusses some ingenious recent approaches, detailing also on possible solutions involving the use of nanomaterials to ensuring the biosensors’ selectivity. Overall, the examples presented illustrate the various tools available when developing enzyme biosensors for new applications and stress the necessity to more comprehensively investigate their selectivity and validate the biosensors versus standard analytical methods.

## 1. Introduction

Selectivity represents the ability of an analytical method to detect the target analyte without being influenced by other sample constituents. It represents one of the key advantages of biosensors, compared to other methods, as they allow to determine an analyte in a complex mixture without resorting to prior separation.

**Figure 1:**
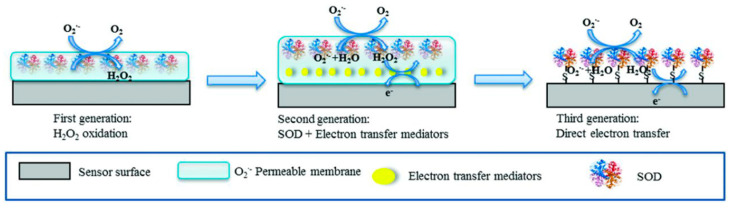
Schematic illustration of first, second and third generation biosensors, exemplified for biosensors based on superoxide dismutase (SOD) for the detection of superoxide anion. Reproduced from [1] with permission.

Enzyme based biosensors hold the largest market share of commercial biosensors and continue to be widely investigated, along with devices based on antibodies, aptamers, cells and other biorecognition elements. Enzymes are biocatalysts, converting their target analyte at high rate. Enzymes are also activated or inhibited by various compounds including pollutants such as pesticides or heavy metals, which provide opportunities for the development of indirect inhibition-based measurements. The selectivity of enzymatic biosensors, whether biocatalytic or inhibition based, is determined by the specificity of the enzyme but the biosensor response is also influenced by design parameters such as: (i) the biosensor type, e.g., first, second or third generation, (ii) the complexity of the sample and (iii) the particularities of the detection method such as the electrode potential, electrode type and surface modification, e.g., in the case of electrochemical measurements. The materials used to create the sensing surface, modifiers, membranes, and the enzyme immobilization method are also contributing to the performance of the biosensors. Developing effective strategies for achieving high selectivity is strongly tied on the type of enzyme biosensor, first, second and third generation, exemplified for superoxide dismutase for the detection of superoxide anion, shown in Figure 1. 

In the first generation biosensors, the reactants or the reaction products are determined electrochemically at high applied potentials, being prone to interferences, which are eliminated by using membranes or sentinel sensors, as detailed below. The second generation biosensors make use of mediators to reduce the overpotential required for detection and thus minimize interferences. For some redox enzymes, direct electron transfer (DET), by ‘wiring‘ or electrically connecting the enzyme with the electrode is enabled, leading to third generation biosensors with inherently higher selectivity.

The recent years witnessed a tremendous progress in the development of enzymatic biosensors as wearable devices for the non-invasive analysis of biomarkers in physiological fluids [2], for example for the detection of glucose, lactate, alcohol and uric acid analysis in sweat [3]. The in vivo analysis is another area that has seen progress in designing electrochemical enzyme biosensors with enhanced selectivity [4,5,6] best illustrated by implantable electrodes for the detection of neurotransmitters in the brain [7]. In addition various biosensors were developed for food analysis, targeting the detection of pesticides, glucose, lactate, glycerol (e.g., for monitoring fermentative processes), biogenic amines (for evaluating the freshness of fish and meat), bisphenol A, etc. In the same time, various enzyme biosensors were reported for monitoring the quality of the environment, for detecting contaminants such as organophosphate and carbamate pesticides, [8] toxic metals such as arsenic [9] or chromium [10], etc. Applications in agriculture or livestock health monitoring have also been reported, with some advanced concepts to interface biosensors with the Internet of Things [11].

While biosensors have widespread applications, a critical question remains: how to achieve accurate and precise measurements and selectively detect analytes in such complex matrices? For example, for implantable biosensors, the list of possible interfering compounds of both endogenous and exogenous origin (e.g., from medication) is extensive, i.e., ascorbic acid, uric acid, acetaminophen, and l-cystine, along with urea, bilirubin, cholesterol, creatinine, dopamine, ephedrine, l-DOPA, methyl-DOPA, glutathione, ibuprofen, gentisic acid, tetracycline, tryglicerides and tolbutamide [12]. Among these, the problem of acetaminophen interference in sensing in biological fluids was longtime recognized, [13] i.e., combining Nafion with cellulose acetate in a composite inner membrane was suggested as a possible solution in implantable glucose biosensors more than 2 decades ago [13]. Acetaminophen interference still appears still appears to be an issue [14,15].

To design a selective enzyme biosensor, the source of possible interferences must first be identified. For example, in electrochemical enzymatic biosensors, electroactive compounds oxidizing or reducing at similar potentials as the target analyte directly influence the analytical signal. In addition, inhibitors, activators or substrates of the enzyme component affect the biosensor signal by influencing the enzyme’s activity, and therefore calibration needs to be performed by taking in account these conditions. The traditional approaches to address the issue of electrochemical interferences are to:

- Use permselective membranes preventing the respective compounds to reach the electrode via charge, size or hydrophobicity-dictated restrictions.
- Integrate a “sentinel” sensor including the same immobilization matrix as the biosensor but lacking the biorecognition element or where the biorecognition element is replaced by an “inert” protein such as bovine serum albumin, BSA. [16] Sentinel sensors record signals due to interfering compounds which are then subtracted from the biosensor’s response.
- Use mediators and redox polymers to lower the applied potential to an ideal potential window where the range of interferences is minimal (ideally close to 0 V); additional opportunities are brought by “wired” enzymes, performing DET.
- Use enzymes to convert the interfering compounds to inactive ones, e.g., ascorbate oxidase to eliminate the interferences due to ascorbate.

With regards to selectivity, limitations introduced by compounds present in the sample which affect enzyme’s activity, were most effectively addressed by multiple (coupled) enzymatic reactions, use of mutant enzymes with altered selectivity or sensor arrays combined with data interpretation using chemometrics. Detailed knowledge of enzyme’s substrate selectivity, knowledge of activators and inhibitors and of the inhibition mechanism by various compounds is paramount to reach the selectivity goals. 

Enzyme’s selectivity profile also depends on the source of enzyme and alterations brought by protein engineering. While some enzymes are specific for target compounds, many have group specificity, which can be useful for large scale screening in food and the environment. Such examples include cholinesterase-inhibiting organophosphorous and carbamate pesticides.

More recent strategies to achieve selective detection with enzyme biosensors use nanomaterials in the enzyme immobilization matrix or as electrode materials. These new advancements are discussed below together with some representative traditional approaches.

## 2. The Innovative Use of Enzyme Kinetic Particularities to Improve the Selectivity

The enzymatic biosensors take advantage of the enzymes’ natural sensitivity and selectivity to achieve fast detection of either the substrate or inhibitor. Nevertheless, there are numerous situations where the enzymes (i) display class selectivity, thus not recognizing a single substrate or (ii) other interfering compounds influence the enzymatic activity by either increasing or decreasing the reaction rates. The magnitude of the issues caused by the partial selectivity displayed by enzymes led to their questioning as being useful analytical devices [17]. There are however some simple solutions to circumvent this lack of selectivity:

- the biosensor could be destined to detect all the recognized compounds and provide the result as a global estimation of all substances present in the sample renouncing to the expectation as being selective.
- the usage of the biosensors is reduced only to samples that are known not the contain the potential interferents or that contain the analyte in huge excess in comparison with the expected level of interfering compounds or more complicated sample pre-treatments and purifications steps are carried out before the actual analysis with the biosensors.

Various strategies can be conceived to improve the analytical performances of enzymatic biosensors, based on the particularities of enzyme kinetics in order to extract more reliable analytical information. These exploitable differences between the enzymes interaction with the substrate/inhibitors/interfering compounds arise from the use of enzymes from different classes/isoforms/origins and the variation of the operational conditions in order to discriminate between analytes presents together in mixtures or to enlarge the spectrum of detectable substances. The design of the improved enzymatic strategies can be based on either (i) parallel enzymatic reactions to combine the information obtained from different independent kinetics set-ups or (ii) successive reactions to further convert the products of the initial reaction in order to eliminate some interferents or to detect compounds that were not directly quantifiable form the sample. 

### 2.1. Employment of Parallel Enzymatic Reactions to Improve Biosensors Performances

The use of several biosensors in parallel aims to extract information from various kinetic conditions, for a better characterization of the sample. The sample is analyzed with each available enzyme/biosensor and from each analysis is obtained a partial result; the combined data allows a better sample characterization. Improved overall analytical results are achieved by taking into consideration the differences between the kinetic properties of various enzymes towards the same analytes, i.e., different affinities, reaction rates, inhibition constants, etc. The combination of parallel enzymatic reactions to increase analytical performances is reviewed in this section.

#### 2.1.1. Use of Substrate Conversion by Multiple Enzymes

##### Alcohols

The biosensors for alcohol detection are based on either alcohol oxidase (AOX) or alcohol dehydrogenase (ADH). These biosensors are typically designed for the analysis of alcoholic beverages. Both enzymes catalyze the oxidation of several alcohols with various affinities. In fact, ADH practically does not recognize methanol; while AOX catalyzes both the oxidation of methanol and ethanol (see reactions (1–2)) but has a significantly higher affinity for methanol in comparison with ethanol [18]. The kinetics of two parallel reactions for two substrates producing a single quantified product (H_2_O_2_) are described according to a Michaelis-Menten theory in the Equations (3)–(8) where the indices 1 and 2 represent methanol and ethanol, respectively. The AOX binds both alcohols and thus the total quantity of the enzyme [E_0_] is equal with the sum of free enzyme [E] and the two formed complexes with methanol [ES^1^] and methanol [ES^2^] (Equation (3)). Thus, the steady state condition takes into consideration both enzyme-substrate complexes (Equation (4) were *k*_1_ represents the forward rate constant, *k*_−1_ represents the reverse rate constant for ES complex and *k*_2_ represents the catalytic rate constant). The partition of the enzyme into two enzyme-substrate complexes influences both the reaction rate for conversion of methanol into formaldehyde (Equation (5)) and ethanol’s conversion into acetaldehyde (Equation (6)). The analytical signal recorded for the AOX biosensor response is based on quantification of hydrogen peroxide (H_2_O_2_) produced in both reactions and it is a sum of both methanol and ethanol conversions (Equation (7)), with a higher influence from methanol. In practice, the AOX analytical signals recorded for methanol-ethanol mixtures in comparison with the signals recorded for solutions containing only one alcohol present alone in the solution is higher than the analytical signals obtained for each individual alcohol and smaller than the sum of the individual signals following the enzyme affinity for each substrate following the specific Michaelis constants *K_M_* and Equation (8). Based on these facts, it is possible to detect both alcohols in mixtures from the interpretation of the analytical signals from ADH and AOX biosensors. Thus, the ethanol was quantified with the ADH biosensor and the methanol was obtained from a complex calibration made by interpolating the AOX responses recorded for a multitude of mixtures methanol-ethanol. This strategy may seem complex, but it extends biosensors’ utility to the screening of counterfeit alcoholic beverages containing the toxic methanol, either alone or mixed with ethanol [19].

(1) $$methanol+O_{2}\overset{AOX}{\to }formaldehyde+H_{2}O_{2}$$

(2) $$ethanol+O_{2}\overset{AOX}{\to }acetaldehyde+H_{2}O_{2}$$

(3) $$\left[E_{0}\right]=\left[E\right]+\left[ES^{1}\right]+\left[ES^{2}\right]$$

(4) $$\begin{array}{ll} \frac{d\left[ES^{1}\right]}{dt}+\frac{d\left[ES^{2}\right]}{dt} & =k_{1}^{1}\left[E\right]\left[S^{1}\right]-k_{-1}^{1}\left[ES^{1}\right]-k_{2}^{1}\left[ES^{1}\right]+k_{1}^{2}\left[E\right]\left[S^{2}\right]-k_{-1}^{2}\left[ES^{2}\right]\\ & -k_{2}^{2}\left[ES^{2}\right]=0 \end{array}$$

(5) $$\frac{d\left[formaldehyde\right]}{dt}=k_{2}^{1}\left[ES^{1}\right]=\frac{V_{max}^{1}\left[S^{1}\right]K_{M}^{2}}{K_{M}^{1}K_{M}^{2}+K_{M}^{2}\left[S^{1}\right]+K_{M}^{1}\left[S^{2}\right]}$$

(6) $$\frac{d\left[acetaldehyde\right]}{dt}=k_{2}^{2}\left[ES^{2}\right]=\frac{V_{max}^{2}\left[S^{2}\right]K_{M}^{1}}{K_{M}^{1}K_{M}^{2}+K_{M}^{2}\left[S^{1}\right]+K_{M}^{1}\left[S^{2}\right]}$$

(7) $$\frac{d\left[H_{2}O_{2}\right]}{dt}=k_{2}^{1}\left[ES^{1}\right]+k_{2}^{2}\left[ES^{2}\right]=\frac{V_{\max}^{1}\left[S^{1}\right]K_{M}^{2}+V_{\max}^{2}\left[S^{2}\right]K_{M}^{1}}{K_{M}^{1}K_{M}^{2}+K_{M}^{2}\left[S^{1}\right]+K_{M}^{1}\left[S^{2}\right]}$$

(8) $$\frac{\frac{d\left[formaldehyde\right]}{dt}}{\frac{d\left[acetaldehyde\right]}{dt}}=\frac{V_{max}^{1}\left[S^{1}\right]K_{M}^{2}}{V_{max}^{2}\left[S^{2}\right]K_{M}^{1}}\Rightarrow \max\Rightarrow K_{M}^{2}>>K_{M}^{1}\;$$

##### Amines

The detection of amines using biosensors benefits from existence of a large number of enzymes that catalyze their oxidation such as: monoamine oxidase, putrescine oxidase, cyclohexylamine oxidase, protein-lysine 6-oxidase, primary-amine oxidase, tyramine oxidase, or diamine oxidase. A representative selection of the substrates recognized by the enzymes is listed in Table 1. A more comprehensive data together with bibliographic citations can be found in the dedicated database [20]. One representative example of biosensors array developed for the resolution of amine mixtures is based on combination of three enzymes with variable substrate specificities, each enzyme having a different preferred substrate for which it displays maximum activity: diamine oxidase (100% putrescine, 50% spermidine, and small responses for spermine and tyramine), primary-amine oxidase (100% histamine, 90% tyramine, and small responses for spermidine, putrescine, cadaverine and spermine) and tyramine oxidase (tyramine is practically the only recognized substrate) as reported by authors [21]. Using the neural network, the best discrimination was achieved for tyramine, followed by histamine, while the poorest results were obtained for putrescine. The study was complemented with an investigation of false positive and false negative results from real food samples validated using chromatographic analysis [21]. Spermine and spermidine are two aliphatic biogenic amines with a relatively similar structure and their selective determination was reported using two engineered enzymes: polyamine oxidase, with selectivity towards both spermine and spermidine (indicated relative responses 100% and 69%, respectively) and spermine oxidase with selectivity towards spermidine (recorded responses to other amines were less than 3%). The analytical signals were used for the analysis of spermine or of the total content of spermine and spermidine from blood samples. The authors reported also the apparent kinetic constants (K_M_^app^, K_cat_^app^, I_max_…) determined by Lineweaver-Burk-type linearization for both enzymes that were immobilized by entrapment in a photopolymerizable matrix on the electrode surface [22], but the application of Michaelis−Menten kinetics is not always suitable for heterogenous systems due to various factors such mass transport [23]. A simpler approach than combining the responses from enzymes with variable responses towards different analytes remains the use of selective enzymes, if available. Thus, putrescine oxidase was used for the specific detection of these putrescine without interferences from other amines such as: spermidine, spermine, cadaverine or tryptamine [24].

##### Phenols

Similar with the amines, the phenols can be oxidized by several enzymes with different substrates affinities and selectivity and combination of these enzymes can be used in order to extract more detailed analytical information on the phenolic content in a sample. Tyrosinase catalyzes two different reactions: (i) monophenolase reaction: one of the bound oxygen atoms is transferred to a monophenol generating an o-diphenol intermediate and (ii) diphenolase reaction, converting *o*-diphenols to *o*-quinone. The tyrosinase is able to recognize both monophenols and *o*-diphenols that are oxidized to quinones. Catechol oxidase is another enzyme that catalyzes the oxidation of a variety of substituted catechols to the corresponding *o*-quinones. Unlike tyrosinase, catechol oxidase cannot catalyze the monooxygenation of monophenols to *o*-diphenols. Laccase is another low specificity enzyme that catalyzes both *o*- and *p*-quinols, and interestingly acts also on aminophenols and phenylenediamine (see Table 1 for a brief listing of some reported substrates, more information being available at the BRENDA enzyme database [20]. 

There are numerous biosensors based on enzymes that catalyze the oxidation of phenols used either as purified preparations or as crude extract from different organisms. Even if the enzymes recognize a large spectrum of substrates, the biosensor response is reported relatively to a chosen analyte [25]. Biosensors that combine different phenol oxidizing enzymes can be designed to (i) enlarge the spectrum of detectable analytes or (ii) the discriminate between different analytes, based on the differences between the analyte-enzyme interactions. In order to extend the analytical performances of these enzymatic biosensors, laccase and tyrosinase were co-immobilized on the same biosensor obtaining an improved Michaelis–Menten kinetic constant when compared with biosensors based on each enzyme [26]. The actual meaning of a combined affinity constant for co-immobilized enzymes is not clear. The co-immobilization of laccase together with tyrosinase has also widened the range of phenolic compounds detected, due to the different catalytic activity of each enzymes, an interesting feature when estimating e.g., the total content of antioxidants found in wine [27]. The exact mechanism responsible for the mechanism of bioamplification in the case of the dual laccase-tyrosinase biosensor was not entirely elucidated. It could be based either on common intermediary compounds from the enzymatic reactions or just on a more effective preservation of enzymatic activity during the immobilization process [28]. Unlike laccase, tyrosinase does not present a significant activity for oxidation of p-/m-benzenediols and related substituted derivatives. Thus, the combined responses due to these two enzymes allow to significantly widen the range of detectable phenolic pollutants with potential applications for measuring the total phenolics in environmental waters. A further increase in the number of detectable phenols was achieved by including in the network a third biosensor based on horseradish peroxidase that is oxidized by hydrogen peroxide and then re-reduced by phenols [29]. In another approach the signals from biosensors based on tyrosinase or laccase combined with responses from non-enzymatic electrodes were interpolated using Artificial Neural Network (ANN) to more precisely discriminate between three analytes, namely catechol, caffeic acid and catechin from mixtures [30].

#### 2.1.2. Detection Based on Signal Reduction (True or Pseudoinhibition)

**Figure 2:**
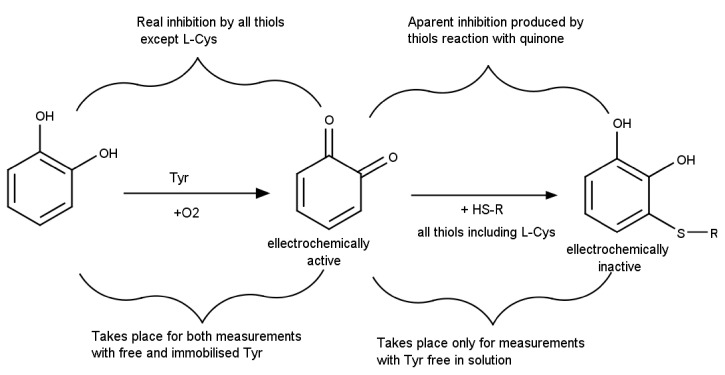
The detection strategy to selectively detect l-Cys without interferences from thiols using tyrosinase (Tyr). Both a classical Tyr inhibition and an analytical signal reduction due to product reaction with thiols are employed.

The classical inhibition of the enzymes leads to a reduction of the analytical signals in electrochemical enzyme biosensors. Additionally, there are cases where chemical reactions of the analyte, interferents or the enzymatic reaction products also lead to a decrease in the analytical signal. While not directly linked to enzymatic activity, this effect is often interpreted and quantified as a “pseudoinhibition”. In this section are presented analytical strategies based on the reduction of analytical signals involving less employed inhibition types or due to other reaction pathways. 

l-Cysteine (l-Cys) is an aminoacid with a thiol moiety that it is difficult to detect using electrochemical methods without interferences from other thiolic compounds that have a similar oxidation potential at bare or mediator-modified electrodes. A selective detection strategy for l-Cys was developed by indirectly quantifying catechol oxidation to quinone catalyzed by tyrosinase (Tyr), followed by quinone reduction. The detection strategy is based on two phenomena: (i) all thiols, including l-Cys, react with catechol, producing electro-inactive adducts leading to an analytical signal decrease and (i) only the interfering thiolic compounds inhibit Tyr while l-Cys does not interact with the enzyme (Figure 2). Thus, while a ‘‘real inhibition’’ corresponds to the enzyme denaturation produced by all thiols excepting l-Cys, there is also an ‘‘apparent inhibition’’ that is measured due to the thiols’ reaction with the electrochemically quantified quinone. The detection strategy uses thus two separate measurements in order to differentiate these two phenomena (i) with the Tyr free in solution together with catechol substrate and thiols compounds, where both real and apparent inhibition take place simultaneously and (ii) with Tyr immobilized on electrode surface that allows separating the enzymatic activity measurement from the incubation with the thiols and thus only the real inhibition can be discerned. The measurements carried out with immobilized Tyr imply three steps in order to avoid contact between the thiols and the quinone: (i) the quantification of the initial enzyme activity in standard catechol solutions, (ii) incubation of the enzymatic biosensor with the sample solution to allow the inhibition to take place and (iii) the quantification of the remnant enzyme activity using (again) standard catechol solutions. For both cases (free and immobilized enzyme), an inhibition percentage was calculated and the differences between the inhibitions were correlated with the l-Cys content. The analytical signals recorded for the two measurement cases have different shapes: (i) linear increase of the current magnitude for Tyr free in solution characteristic to any enzymatic activity measurement and (ii) fast increase to a plateau for Tyr immobilization corresponding to biosensors responses to substrate injection in solution. Due to the differences in the measurement conditions and reaction times, the signal decrease in the case of Tyr free in solution is mainly due to quinone consumption by thiols and not caused by Tyr inhibition. Two thiol compounds with similar as l-Cys were used as model interferents: mercaptoacetic acid and cysteamine. It was observed that both compounds inhibit Tyr and also that mixtures containing the two thiols lead to a larger inhibition percentage than the individual solutions of interfering thiols at the same concentration, but smaller than the sum of individual contributions. This behavior is explained by the parallel competitive reactions. Two practical applications of the method were proposed based on the achieved selectivity for l-Cys: (i) l-Cys selective detection in the presence of other thiolic compounds and (ii) low concentration of thiolic impurities detection from l-Cys-containing products. Both scenarios are relevant for the quality control of pharmaceuticals and nutritional supplements, the utility of the biosensor being demonstrated by analyzing two drugs for the treatment of liver and kidney related conditions [31]. 

Acrolein reacts with thiols leading to an inhibition of the cysteine containing enzymes. Therefore, these enzymes can be used to develop biosensors for the detection of acrolein. Several dehydrogenases were tested were tested and various behaviors were observed: (i) aldehyde dehydrogenase (AlDH) was sensitive to acrolein, but its kinetics were complex by combining inhibition with aldehyde oxidation, (ii) alcohol dehydrogenase (ADH) was inhibited by acrolein and (iii) glutamate dehydrogenase (GDH) was the least inhibited by acrolein. A spectrometric assay measuring the amount of reduced cofactor NADH produced in the enzymatic oxidation of the substrate was developed based on this principle. It was observed that acrolein is a “suicide” substrate for AlDH, thus this enzyme is both inhibited and oxidizes acrolein to acrylic acid even in the presence of its natural substrate, acetaldehyde. The enzymatic reaction rate measured for aldehyde dehydrogenase in the presence of fixed acetaldehyde and increasing acrolein concentrations is initially increasing (two substrates leading to common NADH reaction product, NADH) followed by a decrease (the inhibition by acrolein is becoming predominant). This kinetics prevents the development of analytical assays (same NADH production rate is obtained for two different acrolein concentrations). On the other hand, ADH allowed the detection of acrolein based on enzymatic inhibition in the range 0.2–1 mM. In addition, free l-Cys and glutathione scavenge acrolein and have a protecting effect on enzymes. This natural process was employed to validate the specificity of the enzymatic assay: the contaminated samples of industrial water are mixed with l-Cys and re-measured to discriminate the presence of acrolein from other nonspecific inhibitors [32]. 

Acrylamide is a toxic compound that is naturally detoxified in living organism by coupling with glutathione in a reaction catalyzed by glutathione S-transferase (GST). The activity of glutathione S-transferase is spectrometrically measured using 1-chloro-2,4-dinitrobenzene as color reagent that is enzymatically coupled with glutathione. If the enzymatic reaction medium contains acrylamide (or any other xenobiotic compound recognized by GST) then a part of the glutathione reacts with acrylamide instead of the color reagent. Because both glutathione coupling reactions take place in parallel it is observed a decrease of the color in the enzyme activity measurements, similar with an inhibition. When the concentrations of glutathione and color reagent are kept constant the color variation is dependent only on acrylamide. Thus, pseudo inhibition constants can be calculated since the parallel enzymatic reactions with multiple substrates in competition can be modeled using the competitive inhibition equation, where the inhibition constants *K_I_* is replaced by the ratio between the affinity constants *K_M_* for the competing substrates (see Equation (4) where indices 1 and 2 refer to acrylamide and color reagent, respectively). One can observe that Equation (9) is a particular case of Equation (7) where the second substrate concentration is maintained constant:

(9) $$V_{1}=\frac{V_{max1}\times S_{1}}{K_{M1}+\frac{K_{M1}}{K_{M2}}\times S_{2}+S_{1}}$$

Nevertheless, the affinity constants *K_M_* for the reactions described by Equation (9) are relatively large and a sensitive test for acrylamide cannot be developed based on competitive colorimetric reactions. A simpler approach was developed using the electrochemical monitoring of glutathione depletion in the enzymatic reaction with acrylamide. This strategy has the advantage that it does not require the addition of the 1-chloro-2,4-dinitrobenzene reagent and in consequence is usable also for turbid or colored samples. Equation (5) describes the enzymatic kinetic for a two-substrate reaction, where indices 1 and 2 refer to glutathione and acrylamide, respectively. Of course, if the glutathione concentration is maintained constant, then Equation (10) is simplified to Equation (11) and become the classical Michaelis-Menten relation.

(10) $$V=\frac{V_{max}\times S_{1}\times S_{2}}{K_{1}\times K_{2}+K_{2}\times S_{1}+K_{1}\times S_{2}+S_{1}\times S_{2}}$$

(11) $$V_{2}=\frac{V_{max}^{\prime }\times S_{2}}{K_{M}^{\prime }+S_{2}}$$

The optimum glutathione concentration used was 100 µM, chosen as a compromise between the magnitude of the measured currents, noise and cost (higher for higher glutathione concentration), and the specific enzymatic kinetic conditions. A linear calibration graph from 7 to 50 µM acrylamide and a limit of detection of 5 µM acrylamide were achieved. The method was used for analysis of thermally prepared foods such as potato chips and bread [33].

**Figure 3:**
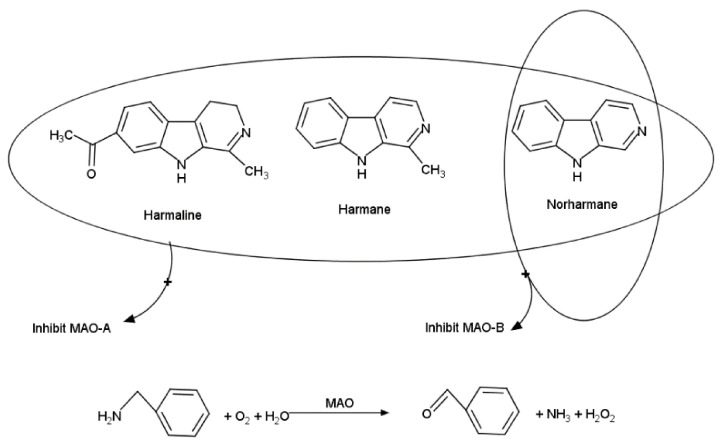
The detection of β-carbolines based on MAO-A global inhibition and discrimination of norharmane based on specific MAO-B inhibition.

Various β-carbolines such as harmane, norharmane, harmaline are found in plants, smoked or thermally prepared foods and their physiological effect is based on the inhibition of mono-amine oxidases (MAO). In fact, there are two isoforms of monoamine oxidase (MAO-A and MAO-B) and they have different inhibition characteristics that are targeted in pharmacology. Thus, MAO-A is inhibited by numerous β-carbolines while MAO-B is inhibited by norharmane and not affected by much higher concentrations of harmane or harmaline (Figure 3). The inhibition for both enzymes is fast reversible and competitive which has two consequences: (i) the same biosensor can be reused for multiple inhibition measurements- a rare feat for enzymatic biosensors based on inhibition that are usually single use and (ii) the substrate concentration influences the observed inhibition percentage. Usually, the inhibition-based biosensors use a large concentration of substrate in order to have a high analytical signal and minimum errors caused by minor changes in substrate concentration. Nevertheless, in the case of fast reversible competitive inhibition, the substrate leads to enzyme reactivation and to a decrease of the recorded inhibition percentages leading to poorer analytical figures of merit. In consequence, the substrate concentration was optimized to reach a “compromise” value as: (i) lower substrate concentrations induce a too small analytical signal for precision measurements even if the inhibition percentage is higher and (ii) higher substrate concentrations reduce the measured inhibition percentage even if the analytical signal is higher. Two biosensors were used for each sample: (i) one based on MAO-A in order to detect the presence of all β-carbolines and (ii) the other based on MAO-B to identify the presence of norharmane in mixtures. The analysis of food samples with the MAOs biosensors provide the results as both content of norharmane from MAO-B biosensor and an evaluation of the overall content of β-carbolines expressed as equivalent norharmane quantity that produce a similar inhibition [34]. The accuracy of the MAO-A and MAO-B biosensors was verified by the analysis of norharmane-spiked samples of unroasted green coffee and smoked fish, for which recoveries of 99.0–110.1% were calculated.

Another example of extracting supplementary analytical information with a system combining two biosensors is based on: (i) an acetylcholinesterase from *Drosophila melanogaster* with E69W mutation to have increased sensitivity towards omethoate, a neurotoxic insecticide and (ii) an acetylcholinesterase extracted from electric eel that is naturally resistant to the insecticide omethoate. The differences between the enzymes are very important, thus it was not observed any inhibition for resistant acetylcholinesterase extracted from electric eel using omethoate solutions with concentrations that induce a complete inhibition of the mutant, sensitive enzyme. With the exception of omethoate, other interfering substances produced a similar inhibition on both enzymes. Thus, if an environmental water sample had an inhibitory effect only on the biosensor based on the mutant sensitive enzyme then omethoate was present, while if both biosensors showed an inhibitory response then other compounds were present in the sample [35].

#### 2.1.3. Applications of Bio E-Tongues Based on Enzyme Biosensors

The bio electronic tongues, or e-tongues, are a special application of parallel reactions where the analytical signal is thoroughly processed using complex chemometric analysis tools (e.g., partial least squares (PLS), principle component regression (PCR), linear discriminant analysis (LDA), artificial neural networks (ANN) or support vector machines (SVM)), provides unique opportunities to enhance the analytical information provided by biosensors by helping characterize, classify and identify ‘true’ signals from cross-responses. These approaches can be applied to discriminate signals obtained from arrays of sensors, or from systems employing enzyme that have limited specificity. The working principle of bio e-tongue devices consisting in an array of several biosensors is based on the fact that enzymes extracted from different organisms or mutant enzymes produced by genetic engineering have different affinities for substances from the same class (either substrates or inhibitors). The combined recorded analytical signals from all biosensors are mathematically interpreted using techniques such as PLS, PCR, PCA or ANNs. These data analysis methods enables discrimination between different analytes, or between analytes and potentially interfering compounds, a feat that is not possible using single conventional biosensing systems [36]. To optimize the sensing-chemometric tool, several sets of standard solutions that contain various concentrations of the analytes in mixture and/or alone are analyzed with the biosensors and the analytical signals are mathematically treated with specialized algorithms that are thus “trained” to be able to indicate the content of unknown samples. There are numerous examples of ANN based on biosensors using acetylcholinesterase inhibition such as discrimination between an organophosphoric insecticide (paraoxon) and a carbamate insecticide (carbofuran) using four different enzymes extracted from electric eel, bovine erythrocytes, rat, and *Drosophila melanogaster* [37]. Further improvement of the chemometric approaches for the inhibition biosensors aimed at reducing the number of necessary enzymes to only three (from electric eel and two different genetically modified enzymes from *Drosophila melanogaster*) for discrimination between paraoxon and dichlorvos from mixtures. This was possible by using enzymes with important relative variations between the inhibition constants *K_I_*, e.g., for dichlorvos the Kis for the electric eel, B1 and B394 mutants were 0.026, 1.9 and 224 µM^−^^1^ min^−^^1^, respectively [38]. A further reduction of the number of required enzymes to only two (acetylcholinesterase wild-type and genetically modified *Drosophila melanogaster*) necessary to discriminate between three insecticides (chlorpyriphos-oxon, chlorfenvinphos, and azinphos-methyl-oxon) was possible due to large variations between the affinities for these inhibitors. Moreover, in this case it was not as usually the inhibition percentage, but the inhibition reaction rate (i.e., the speed of enzymatic activity decrease), to extract more data from the measurements [39]. Besides chemometric data interpretation based on the inhibition of different acetylcholinesterases, ANN based on other enzymes were reported for analyte discrimination. For example, three protein phosphatases (a natural PP2A isolated from human red blood cells, a recombinant PP2A, and a recombinant PP1) were used for the discrimination of LR and YR microcystins from mixtures based on the differences in the toxicity potency of the analyte linked to the constant of inhibition for each protein phosphatase [40]. Nevertheless, the practical utility of such chemometric strategies, which are based on enzymes that all interact in different manner with all the analytes, is limited by the relative large number of necessary measurements, difficulties to extent to a higher number of analytes and the uncertainties encountered in the analysis of unknown samples that may contain other substances from the same class with the analytes or interferents [41].

One additional specific issue of the bio electronic tongues is related to the relatively poor storage stability of some biosensors that leads to lower analytical signals than expected, hence the necessity of re-calibration, more complex for e-tongues. Besides frequently updated calibration and drift correction approaches [42,43], some possible solutions can come from including redundant biosensors, using a model calibrant to assess the functional status of the e-tongue, and more to the point, improving the stability of biosensors by using mutant, stable enzymes and better immobilization methods [44].

**Figure 4:**
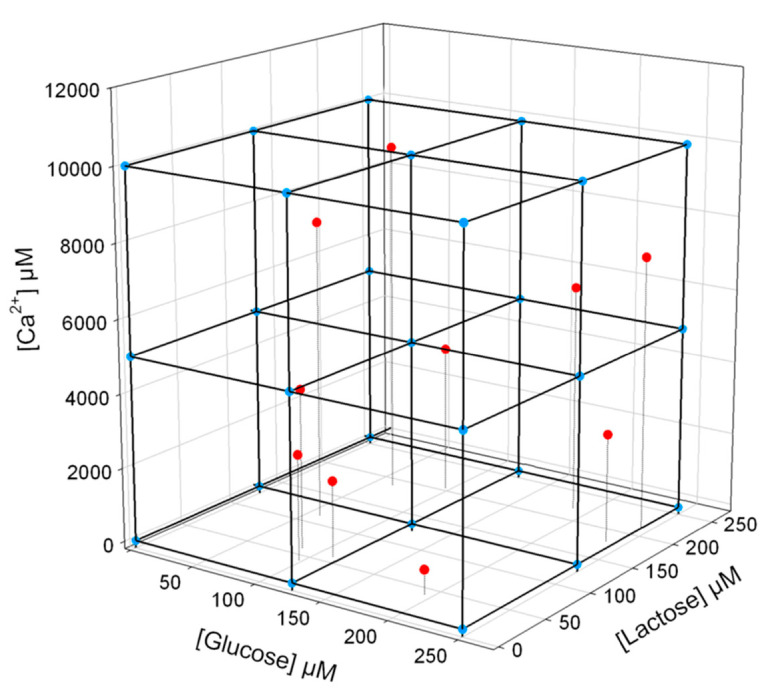
The artificial neural network (ANN) is based on two sets of standard solutions with different concentrations of lactose, glucose, and Ca^2+^ analytes for training (factorial design, blue) and test (random, red), respectively. Reproduced with permission from [45].

Not only the enzyme inhibition was used for development of chemometrically enhanced analytical methods, but also the variations between the catalytic conversion of the substrate was the basis of development of bioelectronic tongue systems. Cellobiose dehydrogenase extracted from various wood degrading fungi (*Myriococcum thermophilum*, *Neurospora crassa* and *Corynascus thermophilus*) have different substrate specificities and were used to discriminate between lactose and glucose in presence of the calcium ions. In addition, the divalent calcium cations at millimolar concentrations were found to enhance the activity of three different cellobiose dehydrogenases for glucose and thus all three analysis of interest for dairy industry were simultaneously detected. The ANN mathematical development is carried out in two stages: a training step with analytical data feed from a sub-set of calibration points followed by other standard solutions used to test the validity of the fitting (Figure 4) before being used for real samples [45].

**Figure 5:**
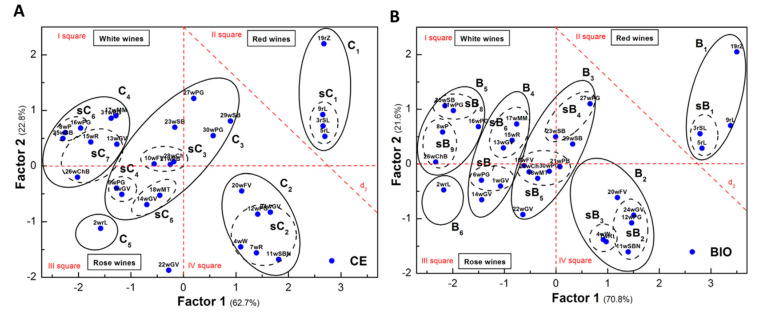
Principal component analysis evaluation of wine samples based on the capillary electrophoresis (CE) (**A**) and biosensor (BIO) analysis of the carboxylic acids (**B**). Reproduced from [46] by permission.

Data obtained from a system using three amperometric biosensors with lactate oxidase, sarcosine oxidase, and fumarase/sarcosine oxidase was processed using principal component analysis (PCA) and self-organized maps (SOM) statistical methods in order to classify 31 wine samples that were also investigated using capillary electrophoresis as a reference method. The analytical signals are based on the estimation of the carboxylic acids that are found in specific ratios in different wines. The malic, citric, succinic, oxaloacetic, acetic, and formic acids competitively inhibit sarcosine oxidase, while lactic acid did not significant inhibit this enzyme and was by consequence quantified with the lactate oxidase biosensor. For a better resolution of carboxylic acids, the sarcosine oxidase biosensor was used alone and also in a variant with co-immobilized fumarase in order to enhance response to tartrate. The chemometric data treatment provided a good resolution of the generated patterns of samples obtaining a good correspondence in the clusters when compared with the capillary electrophoresis (Figure 5) [46].

A network of three deoxynucleoside kinases were used to quantify mixtures of eight pyrimidine nucleosides and nucleoside analogue metabolites used in viral and cancer therapies for point of care patient monitoring. The enzymes were characterized by different catalytic performance (kcat/KM) for each analyte: *Thermotoga maritima* thymidine kinase 1 has exclusive activity for thymidine and its analogues, human 2′-deoxycytidine kinase has complementary activity for 2′-deoxycytidine and its analogues and *Drosophila melanogaster* deoxynucleoside kinase has broad activity covering all investigated substrates. The kinases activity was spectrophotometrically measured and the results were interpolated using Michaelis-Menten equations and Bayesian statistics to identify the probable substrate(s). The mathematical method allowed a very good discrimination between the analytes both alone and in a mixture of two components [47].

Summarizing, it can be said that the use of parallel reactions to analyzing a sample with different biosensors can be conceived by combining: (i) enzymes with high selectivity and enzymes with class recognition in order to discriminate between various analytes or (ii) various enzymes with different kinetic properties towards each analyte and including complex mathematical data treatment for discrimination between the analytes. One practical advantage of the parallel usage of biosensors is the possibility to optimize the reaction condition for each enzyme by providing different working environments such as pH buffers or substrates concentration.

### 2.2. Employment of Successive Enzymatic Reactions to Improve Biosensors Performances

The integration of successive enzymatically catalyzed reactions in the same analytical strategy allows an increased flexibility in the detection mechanisms in order to convert non-detectable analytes, mask interferents and differentially detect different classes of compounds. The combination of the different enzymes may couple: (i) a non-redox enzyme with a redox enzyme to convert electrochemically inactive analytes to detectable products, avoid interferences or discriminate between different classes of compounds or (ii) two or more redox enzymes for an increase of the analytical signal by recycling of the electrochemical active compounds or by amplifying the electrochemical detection. The combination of successive enzymatic cascade reactions used to increase analytical performances is reviewed in this section.

**Figure 6:**
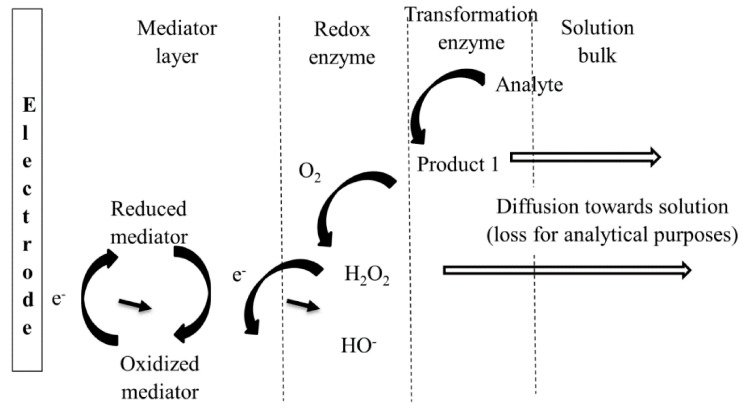
The schematic representation of the working principle of a bienzymatic biosensor based on analyte transformation into a product recognized by a redox enzyme followed by mediated detection of hydrogen peroxide. Note that some of the enzymatic product diffuses towards solution bulk and are lost for analytical purposes.

The kinetic studies of cascade reactions in batch solutions depend on the concentrations of the involved substances/intermediary products and the rate constants for the successive reactions [48]. For enzymatic reactions taking place with enzymes immobilized on electrode surfaces, the kinetics is much more complicated as the local concentrations and reaction rates are influenced by the diffusion of substrates/products and interfacial mechanisms occurring at the electrode surface (e.g., including redox transformation and possible adsorption/desorption) [49]. Ideally, practical biosensors use immobilized enzymes and, in this configuration, both convection and diffusion have important contributions to the cascade reaction kinetics together with the properties of the enzymes that are depending on local configuration and working conditions. Thus, it was observed that for β-galactosidase/glucose oxidase cascade system studied confined in a microchannel the β-galactosidase catalytic reaction showed diffusion control and the glucose oxidase has kinetic control [50]. For analytical applications, the system must be optimized in such a manner that the analytical signal is dependent on the analyte, i.e., the kinetic of the overall process is dependent on the analyte concentration, and the influence of the other steps is minimal. The design of multi-enzymatic biosensor must take into consideration the ratios between the *K_M_* values and the activity of each enzyme for optimum analytical performances. The mathematical modeling of the multi-enzymatic biosensors take into consideration numerous aspects including the enzymatic kinetic properties, mass transport number of layers, diffusion transport [51]. A schematic representation of a biosensor based on cascade enzymatic reaction is presented in Figure 6.

#### 2.2.1. Combination of Redox with Nonredox Enzymes

**Figure 7:**
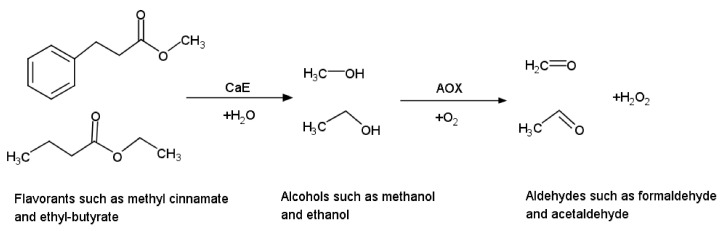
The reactions used for flavorants detection with a bienzymatic biosensor: the flavorants hydrolysis followed by alcohols oxidation and hydrogen peroxide detection.

Numerous classes of nonredox enzymes, such as kinases, transferases, invertases, or hydrolases can be used for the conversion reaction of the analyte to a new product that is subsequently converted by a redox enzyme such as NAD+-dependent dehydrogenase or oxidase in an analytical useful reaction [52]. Below one such example of biosensor is detailed based on cascade reactions obtained by the combination of two enzymes, carboxyl esterase (CaE) and the alcohol oxidase (AOX), to extend the range of substances that are analyzed. Using this system, it was possible to evaluate the total content of ester flavorants in food samples based on two successive enzymatic reactions: (i) hydrolysis of the ester group of the flavorants catalyzed by CaE producing alcohols followed by (ii) alcohols oxidation catalyzed by AOX and finally, the quantification of the produced H_2_O_2_ (Figure 7). Four different flavorants were analyzed: methyl cinnamate, ethyl cinnamate, methyl butyrate and ethyl butyrate. The variability of the flavorants structure implies that CaE has different reaction rates and affinities for each analyte. Two alcohols were released (methanol and ethanol) that are oxidized by AOX following Equation (3) again with different reaction rates and affinities. The combination of both enzymes led to various sensibilities, e.g., the limits of detection being: 0.8 µM methyl butyrate, 2 µM methyl cinnamate, 4 µM ethyl butyrate and 9 µM ethyl cinnamate, respectively and both enzymes influenced the overall response. Thus, methyl containing flavorants are detected at lower concentrations that ethyl based flavorants due to higher affinity of AOX for methanol in comparison with ethanol. Also, the butyrate flavorants are determined at slightly lower concentrations in comparison with cinnamates due to specific CaE substrate affinities. When mixtures of flavorants are present in the same sample, the overall kinetics is even more complicated and for optimum detection a higher activity of CaE in comparison with AOX was used in order to minimize its influence on the measured analytical signal. These biosensors were used for food analysis and the results were quantified by interpolating the response from a single calibration curve (methyl butyrate) since the biosensor was not able to discriminate between different flavorants. Therefore, only an estimation of the global content is possible [53].

Besides the analyte’s conversion to detectable products, the use of cascade enzymatic reactions may be employed for the degradation of some interfering substances or for discriminating between different compounds. There are two related enzymes, aryldialkylphosphatase (EC 3.1.8.1) and diisopropyl-fluorophosphatase (EC 3.1.8.2) that have phosphotriesterase activity [20] (leading to the degradation of organophosphorus toxic compounds (insecticides and nerve gases) that can be combined with acetylcholinesterase in order to better discriminate among classes of inhibitory compounds. One such example has been reported showing the possibility to discriminate between organophosphate and carbamate insecticides based on two acetylcholinesterase inhibition measurements done directly (both organophosphate and carbamate insecticides inhibit) or after sample treatment with phosphotriesterase for 10 min, when only the remaining carbamate insecticides have an inhibitory effect [54]. The detoxifying phosphotriesterase enzyme can be immobilized in a column in a flow system in order to use it multiple times since it is not degraded by the contact with the sample [55] unlike the acetylcholinesterase whose activity decreases during the measurements.

#### 2.2.2. Combination of Multiple Redox Enzymes

The combination of two redox enzymes can be beneficial to improve the electrochemical detection and several bienzymatic strategies have been reported for both oxidase and dehydrogenase enzymes. In the cases of oxidases, horseradish peroxidase is widely used to facilitate the H_2_O_2_ electrochemical detection at low potentials, one typical example being the immobilization of glutamate oxidase on top of a horseradish peroxidase (HRP)/redox polymer layer for the detection glutamic acid from mouse astrocytes [56]. The HRP is an enzyme widely used as a label in ELISA. HRP is also known to intermediate the transfer of electrons between electrode and H_2_O_2_ for a desired improved sensitivity. Nevertheless, the peroxidase usage is not strictly required since there are: (i) very effective electrochemical mediators for H_2_O_2_ such as copper stabilized Prussian blue [34] so efficient that are considered to be “artificial peroxidases” [57], (ii) the possibility to mediate the electrons directly to the some oxidases such as glucose oxidase that can accommodate a variety of oxidants as co-substrates unlike other oxidases such as alcohol oxidase which is a ‘true oxidase’ i.e., selective to dioxygen as the sole acceptable cosubstrate [58] or (iii) even the possibility of direct electron transfer between the electrode and the oxidases [59].

In a similar manner, NAD^+^-dependent dehydrogenases can be combined with diaphorase in an attempt to improve the electrochemical detection. In fact, diaphorase is a vague term that is applied to several different enzymes which catalyze the oxidation of either NADH or NADPH in the presence of an electron acceptor or electrochemical mediator. The dehydrogenase oxidizes the analyte producing the reduced form NAD(P)H and the role of diaphorases is to re-oxidizes NAD(P)H into the oxidized form NAD(P)^+^ that can be reused by the dehydrogenase. Thus, the diaphorases both recycles NAD(P)^+^/NAD(P)H that is useful to shift the equilibrium in the desired direction and also facilitate the electrochemical detection. One typical example of a combination dehydrogenase with diaphorase is the detection of glucose using NAD-dependent glucose dehydrogenase co-immobilized with diaphorase from *Bacillus stearothermophilus* (EC 1.6.99.-) and an osmium complex used as an electrochemical mediator between the electrode and diaphorase [60]. Nevertheless, the advantages provided by diaphorase must be balanced against the inherent complications due to a bienzymatic enzyme and one should also take into consideration the NAD(P)H can be electrochemically detected using various electrode-mediators [61]. One should be also aware that diaphorase is a vague term that is applied to several different enzymes which catalyze the oxidation of either NADH or NADPH in the presence of an electron acceptor or electrochemical mediator. Thus, in BRENDA enzyme database (www.brenda-enzymes.org) [20] there are multiple enzymes that could be considered diaphorases listed with EC numbers: 1.8.1.4, 1.6.99.3, 1.6.99.1, 1.6.5.5, 1.6.5.2, 1.6.2.2, 1.5.1.30 or 1.14.13.39.

As mentioned above, coupled successive reactions are useful to shift the equilibrium in the desired direction for reversible enzymatic reactions. Another example is the detection of lactate using lactate dehydrogenase to oxidize the analyte to pyruvate and NADH. The equilibrium is shifted towards lactate conversion by the addition of a second enzyme, pyruvate oxidase that has the advantage of producing additional ions. This provides the basis of the electrochemical impedance measurements due to change of conductivity [62]. This bienzymatic biosensor was applied to detect lactate in food samples without pyruvate such as yogurts, with good accuracy based on 97.4–107.3% calculated recoveries in spiked samples [62].

### 2.3. Potential Downsides of Combination of Multiple Enzymes

Whenever possible the number of enzymes involved in the cascade reactions strategy should be kept at minimum. For example, triglyceride biosensors are reported by the co-immobilization of three enzymes: lipase (that hydrolyzes the triglyceride to fatty acids and glycerol), glycerol kinase (to convert glycerol to glycerol 3-phosphate using ATP) and finally glycerol-3-phosphate oxidase (for the electrochemical detection based on liberated hydrogen peroxide) [63]. The number of required enzymes was reduced to only two: the lipase and glycerol dehydrogenase (for the specific electrochemical detection of the glycerol produced in the first reaction) [64]. Based on the development of a glassy carbon electrodes modified with copper oxide nanoparticles supported on a multiwalled carbon nanotubes/pectin composite that is suitable for the electrochemical oxidation of glycerol [65], it was possible to reduce the number of enzymes necessary for the triglyceride detection to only lipase since there are no supplementary redox enzymes require for the electrochemical reaction [66]. Another example of a highly complex trienzymatic system for insecticides determination was reported based on acetylcholinesterase inhibition using on a combination of: acetylcholinesterase (that hydrolyses the acetylcholine to choline), choline oxidase (that oxidizes choline producing of H_2_O_2_) and horseradish peroxidase (for electrochemical detection of H_2_O_2_) [67]. A simpler option is to use a bienzymatic format based on acetylcholinesterase coupled with choline oxidase and an electrochemical mediator [68]. Nevertheless, the insecticide detection is dependent on the acetylcholinesterase inhibition and there is the option to make perfectly functional monoenzymatic biosensors by replacing the natural substrate (acetylcholine) with an artificial substrate (acetylthiocholine) that releases thiocholine by enzymatic hydrolysis and thiocoline can be detected electrochemically using appropriate mediators/modified electrodes [69]. The combination of acetylcholinesterase with choline oxidase is clearly necessary in the case of analysis of acetylcholine in biologic samples (e.g., for the diagnosis of Alzheimer’s disease) since in this case the natural substrate and not the acetylcholinesterase activity is the parameter of interest [70].

Another reason to keep the number of enzymes at minimum for cascade reactions is the fact that some enzymes are inhibited by various compounds that could be present in the sample matrix. Thus, the peroxidase that is used to facilitate the electrochemical detection of H_2_O_2_ is inhibited by compounds such as: glyphosate [71], heavy metals [72] or sulfide [73] to such an extent that it was used alone to make inhibition based biosensor for these compounds. Same potential inhibition should be taken into consideration also when using diaphorase for NA(D)PH electrochemistry purposes, this particular situation being even more complex since numerous enzymes are referred under the generic “diaphorase” name and thus the screening of the databases for potential inhibitors is even more complicated. Just to name a few examples, reported inhibitors for diaphorase include: anticoagulant dicoumarol [74], indolequinones derivatives [75] or stilbenes derivatives.

Summarizing, the enzymatic biosensors based on cascade reactions allows (i) the detection of non-redox analytes, (ii) improvement of the analytical figures of merit by combination of more redox enzymes for improve detection or cofactor recycling and (iii) sample treatment to avoid some interferences or improved analyte resolution.

### 2.4. Addressing the Selectivity of Enzymes by Engineering Approaches and Use of Novel Extremo-Philic Enzymes

While several limitations related to their stability and specificity could be encountered for most of enzymes originating from mesophilic organisms, isolation of new candidates from various sources including extreme environments, and applying different protein engineering approaches represent recent strategies for improving the properties of biocatalysts used in biosensing.

#### 2.4.1. Extremozymes

Enzymatic biosensors could lead to a high selectivity for targeted compounds based on their structural features and organism source [76]. Extremophilic enzymes (extremozymes) originating from microorganisms adapted to various extreme environments, in particular the ones characterized by high and low temperatures, high salinity or hydrostatic pressure, have been used for the last decades as potent biocatalysts for a large range of biotechnological and biosensing applications [77,78,79].

A series of thermostable extremozymes from thermophilic bacteria and archaea constituted enhanced catalysts for fluorescent based biosensors. Among these, glucokinase from *Bacillus stearothermophilus* (BSGK) used for continuous glucose detection was stable and active over two weeks at room temperature [80,81]. Alternatively, glucose dehydrogenase (GD) from the thermoacidophilic archaeon *Thermoplasma acidophilum* was employed for selective non-consuming glucose sensing based on the apo-enzyme interaction with 8-anilino-1-naphthalenesulfonic acid, a useful approach in sensing exploiting inactive catalysts in the absence of required cofactors [82].

Selective analysis of organophosphorus agents was performed based on halophiles originating organophosphorus acid anhydrolases (OPAAs; E. C. 3.1.8.2) [83,84]. These extremozymes are highly active when hydrolyzing the P-F bond from the nerve agents soman and sarin [85], while unable to hydrolyze P-O, P-S bonds or P-CN bonds from most OP insecticides [86]. These enzymes provided a selective biorecognition element for monitoring fluorine containing OP compounds [87]. Meanwhile, two archaeal phosphotriesterases from the hyperthermophilic archaea *Sulfolobus solfataricus* and *Sulfolobus acidocaldarius* showed hydrolytic activity against the OP pesticides paraoxon and methyl paraoxon [88].

#### 2.4.2. Protein Engineering Approaches

Protein engineering was recently used as an efficient tool for modifying the stability, activity, substrate specificity and stereoselectivity of enzymes based on site-directed mutagenesis (rational engineering), directed evolution and combined (semi-rational) approaches [89,90]. Using structure–function relationships of corroborated functional characteristics and crystallographic enzyme data, a rational engineering approach considers mutation of specific residues leading to altered kinetics for various substrates [91]. Alternatively, directed evolution imitating the natural evolution process [92] allows the selection of a catalyst variant with enhanced properties from a randomly generated DNA library of targeted genes [93].

The selectivity of a series of electrochemical biosensors was improved when using modified enzymes by site-directed mutagenesis [94]. Acetylcholinesterase (AChE) based sensors responding to organophosphate and carbamate insecticides, besides several toxins, provided specific detection for carbaryl, carbofuran and pirimicarb for the E69W, Y370A and 161V mutants with up to 20-fold enhanced sensitivity [95]. A high sensitivity for the detection of various cyanobacterial neurotoxins was obtained using a broad range of AChE mutants as a result of nucleotide deletion, insertion and replacement [96]. Similarly, protein engineering of AChE from the gastrointestinal nematode *Nippostrongylus brasiliensis* provided a high-yield sensing component for selective detection of insecticides [97].

Altered substrate specificity was also obtained by site directed mutagenesis of residues involved in substrate binding of the *Bacillus* sp. MN chitosanase (E309R and N319E), leading to muteins able to bind N-acetyl-d-glucosamine [98] and unable to hydrolyze the fully deacetylated chito-oligosaccharide tetramer [99]. Moreover, a series of seven mutations (V119D, S262K, N291D, D293T, G319S, D358G, and D368H) induced in alpha-gliadin peptidase by a computational protein design approach that enhanced the number of hydrogen bonds within substrate binding pockets led to an increased specificity for the immunogenic fragments of gluten peptides by 877-fold, with putative application in biosensing [100].

Directed evolution applying random mutagenesis by error-prone PCR (Crum et al., 2016) was used for engineering cyanide degrading nitrilase from *Bacillus pumilus*. Screening the functional properties of the mutants revealed an increased affinity of the D172N mutant for the substrate by 5-fold (0.7 mM) as compared to wild-type enzyme (3.6 mM) in support of the 3-D structural approach as putative tool for selective catalysts [101].

A zinc metallopeptidase neprilysin (NEP), a key enzyme involved in blood pressure regulation and pain nociception processing, was engineered by extensive 2-round site-directed mutagenesis targeting residues from the solvent accessible protein core of the NEP extracellular domain and further selection based on kinetic evaluation and crystallographic structure analysis. This approach generated variants with improved activity and specificity for amyloid beta (Aβ) [102]. The functional characterization of the mutants indicated a specificity increase by 12-fold for the Aβ 1-40 peptide in the case of the G714K, and by 40-fold for the double mutant G399V/G714K [102]

Positive selection was also used for generating enzyme variants with altered functional properties. Alcohol oxidases (AO) mutants from two *Hansenula polymorpha* strains obtained by cultivation in the presence of allyl alcohol and methanol provided modified biocomponents of selective amperometric biosensors for the detection of ethanol [103]. The AO mutant presenting multiple point mutations (I21V, I45V, P148L, K150R, N306D, T527S, F532S, and W567T) showed a 4-fold decreased substrate affinity from Km of 0.62 mM to 2.48 mM without decreasing the maximal velocity [103].

## 3. Effect of the Immobilization Method and Permselective Membranes

### 3.1. Effect of the Immobilization Method and the Potential of Nanomaterials as Immobilization Matrices

Enzyme immobilization affects the biosensor selectivity as the diffusion of various substrates through the immobilization matrix is influenced by the immobilization material, and the hydrophilicity of the microenvironment. Moreover, the immobilization of enzymes on supports might induce steric hindrances for some substrates or inhibitors. Immobilization in redox polymers, or on nanomaterials and functionalized interfaces promotes DET, reducing the applied potential and minimizing interferences. Composite materials where polymers are combined with high conductivity, high area nanomaterials are often an efficient solution for attaching active enzyme to electrodes and obtain high electrochemical signals. This is particularly important in minisaturised sensors, as demonstrated e.g., with graphene nanoplatelets and poly(styrene)-block-poly(acrylic acid) modified electrodes with adsorbed ascorbate oxidase, in a paper based impedimetric assay for detecting ascorbic acid in small volumes (0.4–2 µL) of tear fluid [104].

One illustrative example of how the immobilization affects stereoselectivity is presented by an amperometric biosensor for l- and d-epinephrine, where biotinilated polyphenol oxidase was immobilized via avidin to a sensor covered with a conducting film of biotinilated polycarbazole. The polycarbazole layer presented higher permeability (and therefore the biosensor had an enhanced response) for the d-enantiomer of epinephrine. [105]. In some cases, the immobilization of the biocatalysts lead to an increase in efficiency and even stabilization of the biomolecule for extensive periods of times as compared to the free enzyme. For example, HRP adsorbed on graphene oxide had two times higher efficiency for 2,4-dimetheoxyphenol degradation compared to the free enzyme, while the catalytic efficiency towards other phenolic substrates remained unchanged after enzyme immobilization [106]. This study was aimed at industrial applications, nonetheless the strategy can be exploited also in the biosensing field. In an inhibition-based laccase biosensor for arsenite, oriented immobilization on an electrode modified with anthracene functionalized MWCNT electrodes resulted in minimizing the inhibitory effects of chloride [107].

Redox polymers have multiple uses in biosensors: (i) provide an immobilization matrix that ensures stable attachment and preservation of enzyme’s activity by providing an environment of adequate hydrophilicity, (ii) facilitate the wiring of enzyme to electrodes for direct electron transfer and (iii) act as electrochemical mediators, lowering the potential required for detecting the compound of interest [108]. All these mechanisms affect also the selectivity of the obtained devices. The interest in redox polymers, originates from the possibility to design them in such a way to tailor their formal potential to fit a specific enzyme and application [108,109]. For example, a stable redox polymer consisting in an Os-complex covalently bound to a poly(methacrylate)- backbone had a formal potential around +30 mV versus Ag/AgCl/3 M KCl [110]. At this potential, uric acid and ascorbic acid were not oxidized, and the reduction of O_2_ was not occurring. Immobilization of pyrroloquinoline quinone (PQQ)-dependent glucose dehydrogenase in the redox hydrogel lead to selective biosensors for glucose with apparent Km = 0.98–2.98 mM, depending on the ratio enzyme: redox polymer.

In a different study, HRP was immobilized in a Os-redox polymer at the surface of carbon electrodes, enabling the selective determination of H_2_O_2_ with a detection limit of 1 nM [111]. The biosensor signal was unaffected by ethanol, adenosine diphosphate (ADP) and antimicyn A (an inhibitor of mitochondrial respiration) and facilitated the monitoring of H_2_O_2_ released by mitochondria extracted from *Sacharomyces cerevisiae*. Optimized, low enzyme and polymer loading at the electrode surface minimized the mass-transport limitations leading to high sensitivity and fast response and thus indirectly contributing to the selective analysis of H_2_O_2_. Selectivity to H_2_O_2_ was confirmed by the addition of catalase.

While redox polymers have definite merits in the immobilization of enzymes while concomitantly facilitating fast electron transfer for a sensitive and selective detection, a recent study cautioned on the necessity of a comprehensive evaluation of the biosensor design. It was found that some target analytes can impact the polymeric films, affecting the accuracy and sensitivity of the assay, e.g., phenolic inhibitors of photosystem II (PSII) increased the intensity of the current generated by a redox polymer, used to entrap and electrically wire PSII to electrodes [112].

Oftentimes, the selectivity is achieved by a combination of factors such as enzyme’s substrate specificity, the (direct) electron transfer occurring at a low applied potential minimizing the number of potential interfering compounds and efficient immobilization of the enzyme promoting a high catalytic current for the substrate of interest compared to other compounds in the sample. For example, cellobiose dehydrogenase from *Corynascus thermophilus* (CtCDH) was investigated for the development of third generation glucose biosensors. The enzyme CtCDH was immobilized on screen-printed carbon electrodes (SPCEs), modified or not with carboxylated single or multi- walled carbon nanotubes, by simple adsorption or by adsorption followed by cross-linking with glutaraldehyde or poly(ethyleneglycol) diglycidyl ether (PEDGE) [113]. The enzyme catalyzes the oxidation of glucose at neutral pH and is able of direct electron transfer, which is facilitated by the interdomain electron transfer between its FAD-dehydrogenase and its haem-cytochrome domains. Thus, glucose detection was performed at a low potential of +100 mV versus Ag/AgCl|0.1 M KCl. Nanomaterials such as CNTs ensure the efficient loading of enzyme in a favorable orientation for DET, moreover it was found that the catalytic current was enhanced when PEDGE was used as linker. Altogether it was found that CtCDH-SPCE-SWCNT electrodes where the enzyme was immobilized via PEDGE presented high sensitivity for glucose detection with a detection limit of 10 µmol L^−1^. Additionally, the biosensor response was not affected significantly by the direct electrochemical oxidation of ascorbic acid, uric acid, acetaminophen or by the enzymatic conversion of other substrates of CtCDH in blood such as galactose, xylose, fucose, rhamnose, sucrose, and xylitol. The response for mannose was found to be about a tenth of that for glucose at equal concentration, however considering that in blood the ratio mannose: glucose is about 1:100, [114] the interference from mannose is considered insignificant [113].

### 3.2. Permselective Membranes

To eliminate interferences, permselective membranes are typically deposited on biosensor surface by drop casting, dip coating, spin coating or electrochemical polymerization. The membranes enable the preferential diffusion of analytes based on their size (e.g., cellulose acetate, polyaniline, polypyrrole) or charge (e.g., Nafion, polyethersulfonic acid, polyvinyl pyridine) [115]. The stability and performance of these membranes depends on temperature and humidity, moreover their interference limiting ability is affected by fouling in real-world samples. Multiple membranes are sometimes used to ensure adequate protection against interferences and stable, accurate readings of the biosensor. Most commonly used membranes are. Nafion^®^, polyurethane, chitosan, cellulose acetate, poly(phenylene diamine), poly(2-hydroxyethyl methacrylate) [115].

Most of the time, the biosensor design includes a combination of polymeric membranes to improve the selectivity of detection: e.g., *m*-phenylendiamine/enzyme layer/outer polyurethane coating [116] or Nafion/polyphenylendiamine/ enzyme layer [117]. Polyurethane membranes are typically deposited as an outer layer in biosensors with the role of preventing fouling and ensuring the operational stability of implantable sensors [118], widening the detection range and reducing the dependency on oxygen in oxidase-based biosensors [116].

Films conveniently obtained by electropolymerisation from pyrrole, phenol, phenylenediamine, substituted naphthalenes e.g., 2,3-diaminonaphthalene, 1,5-diamino-naphthalene [119] etc., were used for a long time in biosensors as permselective membranes, to prevent the access of common interfering compounds in biological samples, while enabling the access of the analyte of interest. For example, films obtained from overoxidised pyrrole, phenylendiamine (particularly from *o*- and *m-*phenylendiamine) were preferred in the first-generation glucose oxidase-based biosensors. These were effective for detecting H_2_O_2_ at the surface of Pt electrodes while minimizing e.g., the oxidation of ascorbic acid, uric acid and acetaminophen in serum [120,121]. Similar designs have been adopting for developing a lactate oxidase biosensor for in vitro and in vivo monitoring of lactate during ischemia and reperfusion to assess pathophysiology of tissue hypoxia [122].

Nafion™ is a perfluorosulphonic acid polymer that repels anions like ascorbic acid and uric acid through electrostatic interactions, impeding considerably their diffusion to the sensor surface, while molecules such as hydrogen peroxide are allowed to pass through the membrane. A recent study on the Nafion™’s interference prevention mechanisms in glucose sensing, looked at how ascorbic acid, glycine, urea, acetylsalicylic acid and acetaminophen affect a Nafion™ coating on a glucose sensor surface. Combined cyclic voltammetry, electrochemical impedance spectroscopy and Fourier Transform infrared (FTIR) spectroscopy studies lead to the conclusion that acetaminophen and acetylsalicylic acid were strongly adsorbed at the membrane-liquid interface and inside the Nafion™ layer, which drastically affected subsequent measurements and preventing accurate operation in biological fluids [123].

Chitosan and chitin are natural, linear aminosaccharide polymers obtained from the shells of shellfish. Chitin is composed of *N*-acetyl-d-glucosamine units while chitosan is obtained by the deacetylation of chitin. In addition to being biodegradable and inexpensive, chitin and chitosan have many desirable traits for enzyme immobilization and multiple possibilities for functionalization, due to the presence of amino and hydroxyl groups in their structure. Chitosan is a cationic polyelectrolyte with a pKa of ~6.5 and thus can bind by electrostatic interactions to negatively charged polymers, biomolecules or negatively charged interfaces. It has high metal binding affinity and readily forms gels. Depending on the method by which are obtained, chitin-based materials have different molecular weights, deacetylation degrees and are available as flakes, powders or gels. Chitosan provides a hydrophilic environment where the enzyme activity is preserved. Additionally, it ensures the mechanical stability and good adherence to surfaces of the enzyme layer. Enzyme immobilization in chitosan was achieved by adsorption, encapsulation (e.g., in chitosan beads), cross-linking with various linkers (mainly with glutaradehyde), covalent binding [124], as well as by affinity [125] for various biotechnology, industrial or analytical applications [124].

In biosensors, chitosan was widely used to for enzyme immobilization and to obtain homogeneous preparations of various nanomaterials for depositing uniform active coatings [126]. Chitosan can enhance the selectivity of electrochemical detection by preventing interferences from ascorbic acid at physiological levels enabling in vivo applications, e.g., the detection of serotonin in live zebrafish embryos with chitosan coated carbon fiber microelectrodes [127]. Chitosan was also used to immobilize tyrosinase on a carbon fiber-based microbiosensor that was implanted in vivo to provide real time in measurements of dopamine in the brain of an anesthetized rat [128]. A detection limit of 1 nM dopamine and a linear range between 10 nM to 220 µM with good selectivity against ascorbic acid, uric acid, serotonin, norepinephrine, epinephrine, and 3,4-dihydroxy-l-phenylalanine (l-DOPA) were achieved.

## 4. Specific Selectivity Advantages Conferred by Nanomaterials

### 4.1. Nanomaterials’ Contributing Role to Biosensor Selectivity

Nanomaterials affect the selectivity of biosensors, including enzyme biosensors in various ways:

- increase the sensitivity of electrochemical biosensors, as they are characterized by a high surface area to volume ratio and a good conductivity (thus enabling a high enzyme loading and high electroactive area). As a consequence the improvement in selectivity is promoted by the enhanced sensitivity [129].
- can electrically connect (“wire”) the enzyme to an electrode, promoting DET from/to the enzyme active center. Examples include single walled carbon nanotubes promoting DET of cellobiose dehydrogenase from *Corynascus thermophilus*, [113] AuNPs [130] or PANI nanotubes [131] for glucose oxidase, zinc oxide nanodisks for superoxide dismutase [132], tungsten oxide (WO_3_) NPs for cytochrome C nitrite reductase [133] etc.
- enable the attachment of mediators [134].
- lower the overpotential required for the detection using dehydrogenases [135] or oxidases [136], thus influencing the selectivity of the assay. The electron transfer rate of some interfering species might be either promoted or slowed down in the same time.
- promote the controlled, oriented immobilization of the enzyme by themselves or after modification with functional groups, e.g., Ni-NTA NPs used for immobilizing histidine-tagged enzymes, Au NPs for attaching enzymes with an engineered cysteine tail, anthracene-functionalized MWCNT for the oriented immobilization of laccase, significantly decreasing enzyme’s inhibition by chloride ions [107] etc.
- promote the quantitative, immobilization of enzymes with preservation of their activity by various strategies and enhanced enzymatic activity. Various such nanobiocomposites have been developed and can be used as materials to improve performance of in biosensors [129,137].
- provide an oxygen reservoir enabling oxidase-based biosensors for performing adequately in oxygen deprived media (e.g., ceria nanoparticles) [138,139].

The role of nanomaterials in enzyme biosensors has been recently reviewed-among other works- in the review of Teymourian et al., [140] discussing specific advantages for first, second and third generation glucose biosensors. The functional properties of nanomaterials-enzyme conjugates and their use to design enzyme-based biosensors have been described with focus on fabrication methods, including screen-printing, ink-jet and 3D printing methods [141].

Hybrid materials based on graphene, carbon nanofibers or carbon nanotubes which also include metal nanoparticles have the ability to reduce the potential required for H_2_O_2_ detection and enhanced the detection sensitivity in first generation glucose biosensors, as Pt or Pd NPs had a contributing electrocatalytic effect to the electrooxidation of H_2_O_2_ [142,143,144]. In the second generation biosensors various nanomaterials and composites including reduced graphene oxide, [145] carbon nanotubes, [146] mesoporous carbon nanoparticles [147] enabled the stable attachment of mediators, thus facilitating the sensitive detection of glucose at lower overpotentials. Oftentimes, the nanomaterials also served for the stable and effective immobilization of GOx, improving the output of the overall catalytic process. Additionally, nanostructured redox hydrogels [148] or materials such as Au nanoclusters functioned as redox mediators [149,150]. By binding AuNPs close (at 13.8 A°) to the FAD redox center of a mutant GOx, the enzyme was effectively “wired” to the electrodes, [130] while in other works PANI nanotubes [131] or a hollow sphere nanostructured composite of poly(3,4-ethylenedioxythiophene) (PEDOT) and NiO [151] enabled the direct electron shuttling from the FAD center of GOx to the electrode surface. In these third generation biosensors, the electrocatalysis of glucose oxidation in the absence of oxygen occurred at −0.3–0.4 V, where ascorbic acid (AA) and uric acid (UA) are not interfering.

### 4.2. Challenges and Perspectives for Nanomaterials in Enzyme Biosensors

As new nanomaterials and nanocomposites are continuously researched, their electrocatalytic abilities for various applications are yet to be discovered. Most studies on new materials include a superficial evaluation of their performance and selectivity in real applications [152]. A limited number of possible interfering compounds were considered and they were studied almost exclusively in buffer solution.

The high surface area and electrocatalytic properties can lead to additional problems such the adsorption of unwanted compounds from the sample solution or the electrocatalysis of other sample components which might be favored in a similar manner as for the analyte of interest [140]. Consequently, the selectivity of these materials should be carefully evaluated and should include a study of all relevant molecules existing in the composition of targeted sample matrix, at typical concentrations.

Considering the progress in designing enzyme nanocomposite materials with largely enhanced activities for biocatalytic applications [137] it is conceivable that some concepts will be adopted in biosensors as well. This presumably will serve to improve selectivity by magnifying the response for the target analyte compared to interfering compounds, yet this remains to be determined. Porous nanomaterials, when combined with enzyme can add selectivity by favoring the diffusion of certain analytes or ensuring a tortuous, longer trajectory of gaseous analytes for a longer interaction time with the substrate. Finally, one exciting area awaiting exploration is coupling the stereoselectivity of some nanomaterials with the attributes of achiral enzymes which would add a new dimension for addressing the enzyme biosensors’ selectivity.

## 5. Modulating the Selectivity by the Particularities of the Detection Method

To address selectivity, the biosensor device and the detection method were adapted to isolate and eliminate interferences. The contribution to the analytical signal due to electrochemically active interferents was most frequently isolated by using sentinel, or ‘control’ electrodes [16] whose signal was subtracted from the biosensor signal. Alternatively, some known interfering compounds such as ascorbic acid or acetaminophen were converted to inactive compounds using additional enzymes such as ascorbate oxidase and polyphenol oxidase, respectively to avoid interferences due to their direct electrochemical oxidation on the biosensor surface. In general, these approaches were combined with the use of permselective membranes to achieve the required selectivity.

For example, in a biosensor aiming to monitor the release of glutamate in the brain, glutamate oxidase was mixed with chitosan at the surface on an Pt microelectrode modified with ascorbate oxidase in a matrix of bovine serum albumin (BSA). The microelectrode was covered with an electropolymerised membrane of poly-*o*-phenylenediamine and the detection of glutamate was performed at +0.6 V vs. Ag/AgCl. Despite the high potential, ascorbic acid did not interfere, not did serotonin, adenosine, dopamine, glucose and uric acid at physiologically relevant concentrations [121]. In another biosensor, based on glucose oxidase, the interferences in the detection of glucose caused by acetaminophen, ascorbic acid and uric acid were reduced to a fourth by including in the biosensor design polyphenoloxidase together with Nafion™ [153].

Self-referencing devices with appropriate signal subtraction procedures enabled operation in complex matrices, including in brain, tears etc. The detection of choline in the rat brain was achieved with a twisted pair of 50 µm diameter Pt/Ir wires corresponding to the biosensor and the sentinel sensor [133]. The biosensor was modified with Nafion™ and choline oxidase (ChOx) cross-linked in chitosan with benzoquinone and the sentinel was developed similarly, except that the enzyme was replaced by BSA. The accuracy of the amperometric detection at 0.7 V vs. Ag/AgCl was not affected by UA and AA, due to the Nafion™ coating. While the biosensor’s response to dopamine was similar to that for choline, it was subtracted through the sentinel electrode. The authors attributed the success of this strategy to the design of the platform where the biosensor and the sentinel were placed side-by-side and to the correction of the sentinel signal (in phase and amplitude, over a range of frequencies to match the biosensor signal), before its subtraction [133].

Another example of selective detection enabled by sentinel sensor is a contact lens biosensor for glucose monitoring. Tears contain water, electrolytes (sodium, potassium, chloride, bicarbonate, magnesium, and calcium), proteins (lysozyme, lactoferrin, lipocalin, and IgA), lipids, mucins, defensins and small molecules including glucose, urea, lactate [154]. In the contact lens biosensor, the selective detection system included an active biosensor where GOx was immobilized in a titania sol-gel and the sentinel sensor, obtained in the same manner but with deactivated enzyme [155].

**Figure 8:**
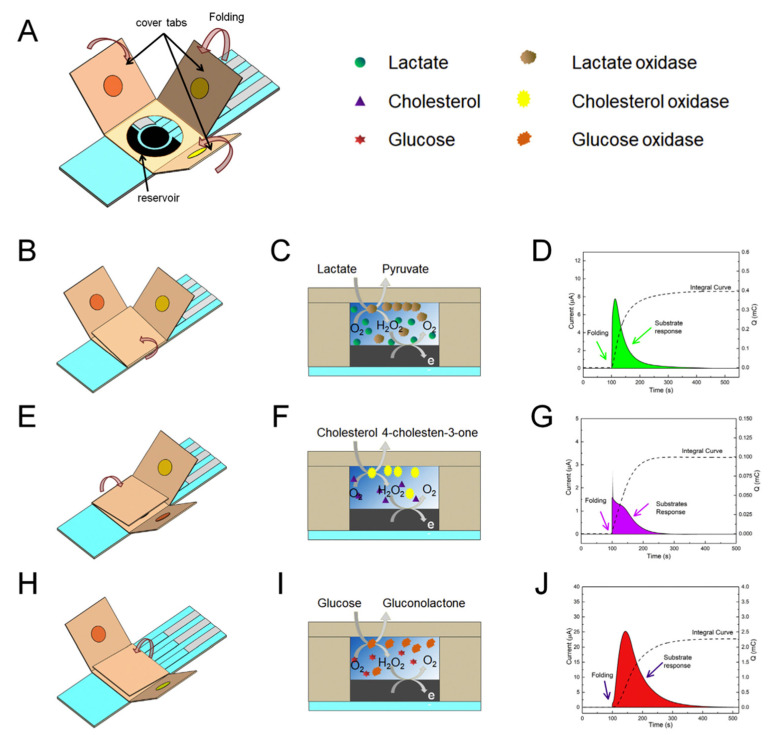
Scheme of the origami-based biosensor. (**A**): General structure of the origami based biosensor. (**B**,**E**,**H**): Folding scheme for the detection of lactate, cholesterol and glucose. (**C**,**F**,**I**): Schematic illustration of the transformations involved in the electrocatalytic determination of lactate, cholesterol and glucose. (**D**,**G**,**J**): The curves of current versus time (solid lines) and integrated charge over time recorded with the biosensor for the detection of lactate, cholesterol and glucose. Reproduced from [157] with permission.

More recently, origami type paper devices separating the contributions from electrochemically active interferents and from the substrate of interest were proposed as a generic solution with large applicability to ensure the selectivity in electrochemical enzymatic biosensors [156,157]. The principle for eliminating the interferences is based on combining a screen-printed electrode with a foldable, enzyme loaded origami paper and on performing the electrolysis of the sample before the enzymatic reaction [156]. As illustrated in a biosensor for the detection of lactate, glucose and cholesterol in serum [157], the T-shaped origami paper has a hole in the centre which delimitates a sample well when fixed on the screen-printed Pt electrode (Figure 8A). The three side “cover tabs” are preloaded each with a different enzyme, e.g., lactate, cholesterol and glucose oxidase. In the first step, after placing the sample in the well (a total volume of 6.5 µL sample in the 7 mm wide well, to cover all three electrodes with a thin layer of sample), the electroactive compounds were eliminated by electrolysis at the applied potential of 0.7 V vs. Ag/AgCl. This took about 100 s [157]. Next, one of the cover tabs was folded over the sample well, the respective enzyme dissolved in the sample enabling a specific catalytic transformation, leading to the production of H_2_O_2._ The H_2_O_2_ was immediately oxidized on the surface of the screen-printed Pt electrode and detected by coulometry (Figure 8).

A note should be made here that sentinel devices or origami paper will not eliminate interferences from non-electroactive sample components which are either substrates, inhibitors or activators of the enzyme component of the biosensor. Multi-sensor arrays combined with chemometric analysis (bio e-tongues) are more suitable approaches in this case.

## 6. Solving Challenges in Real Samples: Selectivity Improvement for Superoxide Anion Detection

The superoxide radical anion (O_2_^•−^) is the primary species produced during the oxidative stress, a process related to disturbances in cellular processes in plants, bacteria, animals and in the human body. Oxidative stress is linked to a plethora of medical conditions, including cancer. Superoxide dissociates fast in aqueous solutions, with a half-life lower than 50 milliseconds, depending on the composition of the surrounding medium and has a high diffusion rate. [158] Moreover, its intracellular concentration in the human body covers a wide range, rising from the normal levels of 10–100 nM up to 0.1 mM during extreme oxidative stress or in severe diseases [159].

Being a primary ROS, O_2_^•−^ is as a biologically relevant target in tests aiming at the evaluation of the antioxidant, radical scavenging effect of individual compounds, food or beverages. The detection of superoxide radical can be accomplished by various methods including electron spin resonance, high performance liquid chromatography coupled with mass–spectrometry, optical (mainly spectrophotometry and fluorescence) and electrochemical procedures [1,159].

The sensitive, selective and real time monitoring of O_2_^•−^ in situ, e.g., in living cells was most appropriately addressed by fluorescence and electrochemical methods. While very powerful for imaging and useful for studying cellular processes, fluorescent methods have also some drawbacks related to the cell permeability, stability and the toxicity of the probes. The cost, complexity of equipment, and the difficulty in achieving real-time detection pose limitations for applying fluorescence based methods in some applications, e.g., screening of antioxidants.

Electrochemical sensors presume simpler, lower cost equipment and overcome some of the above cited problems encountered with fluorescence probes. The selective electrochemical detection of superoxide anion was achieved with biosensors modified with enzymes, complexes and nanocomposites acting as enzyme mimics or with carbon, Au or platinized electrodes, either bare or modified with polymers or nanomaterials. Two types of enzymatic biosensors have been reported for the detection of superoxide anion (O_2_^•−^) using either cytochrome C (cyt C) and superoxide dismutase (SOD).

Cyt C biosensors measure O_2_^•−^ based on the reduction—by O_2_^•−^—of the heme redox center in the enzyme, followed by the electrochemical re-oxidation of cytochrome C at the electrode surface at an appropriate potential. The direct electron transfer between cyt C and the electrode is facilitated by the accessibility of the heme centre, which is not buried deep inside the protein. The generated anodic current is proportional to the concentration of O_2_^•−^ in the medium. Based on this principle various biosensors for O_2_^•−^ detection were developed for applications ranging from monitoring oxidative burst in renal cell cultures exposed to calcium oxalate [160] to evaluating the antioxidant capacity of foods [161,162]. Attaching cyt C to SAM of thiols on Au electrodes is one of preferred strategies for developing biosensors for O_2_^•−^ detection [160]. The usefulness of cyt C in these biosensors was questioned, since the direct and cyt C catalyzed oxidation occur at very close potentials. In biosensors where cyt C was covalently attached to a short SAM of 3,3-dithiopropionic acid, up to 70% of the signal came from the direct oxidation of O_2_^•−^ [163]. Nevertheless, it was also shown that the direct detection of O_2_^•−^ on SAM modified electrodes is affected by interferences from ascorbic acid and H_2_O_2_, while cyt C-mediated detection provided better selectivity and protection against non-specific adsorption [164]. Later the biosensor was applied to monitor ROS released species and antioxidant effects of nanoparticles during ischemia-reperfusion induced injury in infectious colitis [165].

The selective monitoring of O_2_^•^^−^ radicals production in slices of rat brain was reported with Au electrodes modified with mixed SAMs, made from short carboxyl ended thiols such as 3-mercapto-1-propionic acid, enabling the covalent immobilization of cyt C close to electrode surface combined with longer, hydroxyl-terminated thiols (e.g., 11-mercapto-1-undecanol) to prevent non-specific adsorption [166]. The selectivity of such an amperometric biosensor operating at a low potential, 0.15 V was demonstrated by the lack of response towards the potential interfering uric acid and hydrogen peroxide, and was confirmed by the total signal suppression upon addition of SOD [166].

Besides cyt C biosensors, the detection of O_2_^•−^ was achieved using superoxide dismutase (SOD), based on various first, second and third generation biosensor designs, reviewed among others in [1,167].

SOD catalyzes the dismutation of O_2_^•−^ in oxygen and H_2_O_2_ and was shown to perform DET when fixed on certain types of functionalized electrodes. Both the SOD mediated reduction of superoxide to H_2_O_2_ described by Equation (12) below and the oxidation of O_2_^•−^ to O_2_ (Equation 13) can thus be linked to electron transfer by/to an electrode polarized at a convenient potential, to revert to the original state of enzyme’s redox centre and thus determine O_2_^•−^ with high sensitivity.

SOD(Cu (I)) + O_2_^•−^ + 2H+ → SOD(Cu (II)) + H_2_O_2_ (12)

SOD(Cu (II)) + O_2_^•−^ → SOD(Cu (I)) + O_2_ (13)

Since the enzyme catalyzes both an oxidation and a reduction process, there is embedded flexibility in SOD biosensors to choose either the anodic or cathodic detection of O_2_^•−^, depending on which strategy is better for minimizing interferences.

Different approaches have been used to address the selectivity challenge, depending on the biosensor type and design. SOD based biosensors were successfully applied for the detection of O_2_^•−^ in real matrices such as cell cultures [168] (to monitor the release of O_2_^•−^ in relation to the mechanism of cellular processes) and food matrices (e.g., for assessing the antioxidant capacity of beverages [169].

**Figure 9:**
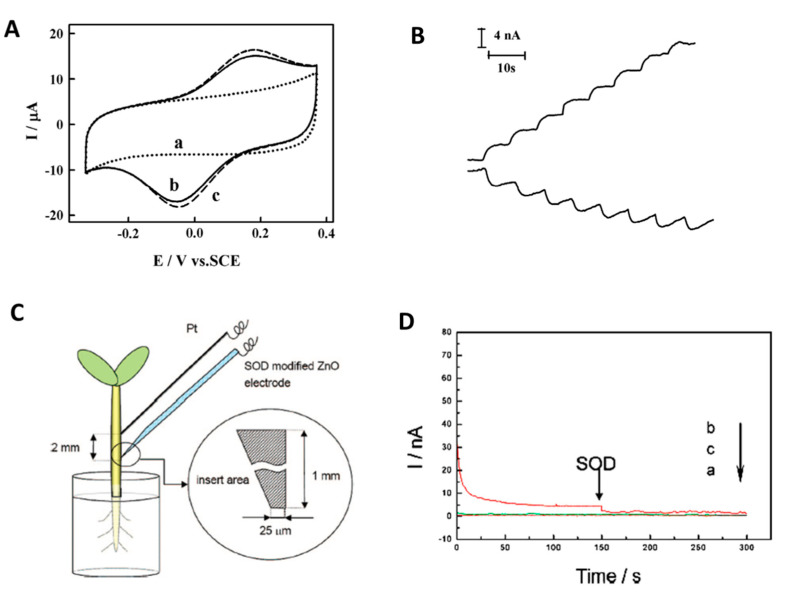
(**A**): CVs recorded with the cys/DenAu/GC electrode in the presence of 100 µM O_2_^•−^ (a) and with the SOD/cys/DenAu/GC electrode in the absence (b) and presence (c) of 100 µM O_2_^•−^ in 25 mM PBS, pH 7.2. (**B**): Amperometric responses of the SOD/cys/DenAu/GC biosensor to successive addition of 0.1 µM O_2_^•−^ at +0.3 V and −0.2 V in 25 mM PBS pH 7.2. (**C**): Schematic representation of the setup for the determination of O_2_^•−^ in bean sprouts with a SOD biosensor. (**D**): Amperometric responses of the SOD/ZnO biosensor (a, b) and ZnO microelectrode at 0.5 vs. Pt in a bean sprout grown under normal atmosphere (a) and in hypoxic conditions (b,c). Reproduced from [172] (**A**,**B**) and [132] (**C**,**D**) by permission.

In first generation biosensors, which targeted the detection of the H_2_O_2_ formed in the enzymatic reaction, the interferences occurring at the high operating potential required for the oxidation of H_2_O_2_ were minimized using different permselective membranes or by self-referencing [1]. Second generation biosensors employed mediators for a more efficient transfer from the redox center of the enzyme to electrode surface. This ensured adequate selectivity for practical applications such as e.g., monitoring the release of O_2_^•−^ from the heart tissue of a Wistar rat. with a biosensor inserted in a flow system. To induce the oxidative stress. endotoxin was administered to the rat prior to the experiment [170]. Third generation SOD biosensors rely on the direct electron transfer from the redox center of SOD to electrode surface, occurring at low potentials, thus promoting the selective detection of O_2_^•−^ in real matrices. The electrochemistry of different types of SOD enzymes was well studied. In conditions promoting DET, SOD enzymes show a pair of redox peaks and a formal potential of 0.04–0.2 V vs. Ag/AgCl [132]. Self-assembled monolayers or various nanomaterials, electrodeposited gold nanostructures shaped as spheres, pyramids, rods [171] or dendrites [172], zinc oxide nanodisks [132], etc., facilitate the attachment of SOD at the electrode surface in an orientation favorable for efficient electron transfer. For example, immobilization of SOD on a glassy carbon electrode modified with dendritic gold and cysteine lead to observe a couple of redox peaks, indicating DET (Figure 9A, curve b). In conditions where the direct detection of O_2_^•−^ did not occur (Figure 9A, curve a), addition of O_2_^•−^ in the sample lead to enhanced height of the anodic and cathodic peaks, based on the transformations in Equations (12)–(13) above followed by electrochemical regeneration of the original state of the enzyme (Figure 9A). Consequently, O_2_^•−^ was detected with similar sensitivity by amperometry at anodic (+0.3 V) and cathodic (−0.2 V) potentials (Figure 9B). Similarly, the copper, zinc-superoxide dismutase (Cu, Zn-SOD), strongly adsorbed by electrostatic interactions on a zinc oxide nanodisks-modified ITO microelectrode was able of DET. The biosensor thus obtained enabled the detection of O_2_^•−^ in the cathodic mode at 0 V where H_2_O_2_, uric acid, ascorbic acid and 3,4-dihydroxyphenylacetic acid were not interfering [132]. The biosensor, together with a Pt microelectrode as a counter/reference, both with a conical tip was inserted about 1 mm in bean sprouts grown under hypoxic conditions (Figure 9C) and the level of O_2_^•−^ was monitored in vivo for 6 days. Control experiments were performed in normal conditions, in the absence of induced oxidative stress. Compared to controls, the electrical current measured at −0.5 vs. Pt (−0.03 V vs. Ag/AgCl) was higher (Figure 9D). To confirm that the measured current originated indeed from the reduction of O_2_^•−^, mediated by the enzyme component in the biosensor, a solution of SOD was injected in the bean sprout in the area close to the biosensor. The signal decreased upon adding SOD, down to a level similar to the one recorded in the absence of the oxidative stress. Additional control experiments with a ZnO nanodisk modified sensor lacking SOD confirmed the lack of direct electrochemical reduction of s O_2_^•−^ in the given experimental conditions.

This study provides a nice illustration of the usefulness of enzyme biosensors and the opportunities for elegant yet simple and effective design for selective in vivo monitoring.

As previously mentioned, besides cyt C and SOD, enzyme mimetic compounds including manganese porphyin complexes [173] or iron porphyrin complex polymers [174], nanomaterials and nanocomposites such nano-Mn_3_(PO_4_)_2_-chitosan, [175], Co-based nanocomposites containing Co_3_(PO_4_)_2_, Co(PO_3_)_2_, Co_2_(OH)PO_4_ [176] hollow or porous carbon nanomaterials derived from zeolitic imidazolate framework-8 [177] have been tested, indicating their potential as highly effective catalysts for the real-time detection of O_2_^•−^ release from living cells [1].

Platinized C, bare C electrodes and Au [178], and more recently single core–shell nanowire electrodes, [179] graphene foam modified with Pt nanoparticles [180] were used by Amatore’s group and others for measuring O_2_^•−^ and other ROS/RNS in various types of living cells. Such sensors, avoiding the stability problems of enzyme biosensors and their strong dependence of pH and temperature, appear as very advantageous. Yet, as shown by the example discussed above, there are still ample opportunities for enzyme biosensors for applications requiring equally high sensitivity and selectivity. Through clever design, nanomaterials may promote an even enhanced performance of such biosensors. Mutant enzymes with increased sensitivity and preserved high selectivity [181] as compared to the wild type proteins may offer yet another competitive advantage.

## 7. Conclusions and Perspectives

Selectivity is one of the critical challenges that need to be overcome when developing practical enzyme biosensors. While the strategies to address this critical issue were refined in the recent years, the basic tools for achieving selective detection remain the same: “know” your sample composition as much as possible; optimize sample preparation to eliminate interfering compounds, design the biosensor and select the operational conditions with interferences in mind, test the selectivity extensively and finally, validate the method by comparing with a standard method. With regards to biosensor design, significant advances have been made and are expected in the future from the design of self-powered biosensors and biofuel cells, where concepts exploiting DET, mutant enzymes with more efficient electron transfer, new materials for achieving improved stability, controlled immobilization and better sensitivity will continue to emerge.

Developing enzyme-based wearables is one area where exciting developments are anticipated in the coming years, through the integration of smart engineering, flexible and autonomous devices. New approaches in achieving stabilization of enzymes on flexible substrates such as flexible displays and textiles, for non-invasive biosensing of biological samples like tears, sweat and saliva, and their integration with wireless and bluetooth signal transmission and the growing internet of things as the basis for digital health, are also expected which can radically change the diagnostics field. Some of these concepts presuming no or minimal sample preparation yet enabling selective detection can also be adapted in the fields of food and environment analysis, for example to create “smart labels” to measure degradation processes in real time.

Many biosensing devices reported in literature are still in the proof of concept stage and most require validation and large scale testing on real samples before demonstrating their potential for real world applications. Sample preparation and biosensor validation, unfortunately are superficially treated in many studies who restrict their work to standard solutions. Looking forward, more concentrated efforts are needed to demonstrate their performance, and confirm selectivity in relevant environments. For these devices to reach their full potential collaboration with practitioners in their fields of use, e.g., clinicians, environmental and food quality experts, would be highly beneficial to advance their development and translation from bench to market. Validation of their capabilities should be demonstrated side-by-side and in conjunction with currently used technologies, and in some cases, combination with other methods might be required to achieve the targeted sensitivities. For example, combining electrochemical detection with complementary optical methods (SPR [182], FTIR [123], SERS [183] etc.) brings more details on the selectivity and the mechanism of the detection, helping to optimize the biosensor design and improve performance. Moreover, to demonstrate accuracy of the developed biosensor the analysis of several real-world samples containing the target analyte at different concentrations, should be performed in parallel with the biosensor and by a standard method. The standard additions method used for calibration and detection compensates for the matrix effect on sensor sensitivity; however the bias introduced by the presence of other electrochemically active compounds to which the sensor responds remain unaccounted for in this method. In such case, the dual-sensor approach (sentinel, sensors, self-referencing) or concepts such as the origami paper, separating the contribution from the target analyte and interfering compounds should be adopted to account for the interferences from the sample. Recent developments in data analytics, artificial intelligence (AI) and computational design enabling more sophisticated signal recognition, background subtraction and the mathematical modeling of sensor design will see continued efforts, which can improve selectivity.

Exploiting enzymes from new sources or mutant enzymes, or taking advantages in the differences in the mechanism of inhibition, using arrays of biosensors or hybrid platforms including also either some chemical sensors, or sensors based on newly developed artificial mimetic elements, provide a broad array of tools that can be used in the future to refine the capabilities of enzyme-based biosensors devices. The development of high performance (bio) e-tongues is sustained by the intensified application of pattern recognition techniques and by the emergence of AI applications in sensing. Nonetheless, their commercial success depends on the proper maintaince of the chemometric model to account for the variations in system’s stability and operational conditions. Last but not least, given the many ways in which nanomaterials can improve the performance of enzyme biosensors, the coming years will undoubtedly bring exciting innovations from the use of nanobiocomposites of enzymes and nanomaterials covering enzyme immobilisation for stability and activity enhancement, an additional dimension in selectivity and synergetic activity of enzyme-nanozyme combinations.

## Figures and Tables

**Table 1:** A selection of reported substrates for different available amine and phenol oxidases compiled from BRENDA enzyme database (www.brenda-enzymes.org, accessed on 28 March 2021) [20]. Benzylamine is used as artificial substrate for measurement of the activity for several oxidases, hence its wide presence.

Recommended Name (Synonyms)	EC Number	Some of the Natural and Other Reported Substrates
monoamine oxidase	1.4.3.4	benzylamine, DOPA, epinephrine, histamine, noradrenaline, serotonin, tryptamine, 4-tyramine, phenylethylamine; it can oxidize secondary and tertiary amines but not methylamine;
primary-amine oxidase (copper-containing monoamine oxidase, plasma amine oxidase)	1.4.3.21	benzylamine, ethylamine, putrescine, cadaverine, cysteamine, spermine, spermidine, spermine, serotonin, tyramine, 2-phenylethylamine; It oxidize primary monoamines and have little or no activity towards diamines or secondary and tertiary amines
diamine oxidase	1.4.3.22	benzylamine, cadaverine, putrescine, spermidine, tyramine, DOPA, cystamine, histamine, diaminopropane, diaminobutane; it oxidizes diamines and some primary monoamines, but have little or no activity towards secondary and tertiary amines
putrescine oxidase (adenine dinucleotide-containing putrescine oxidase)	1.4.3.10	putrescine, 2-hydroxyputrescine
cyclohexylamine oxidase	1.4.3.12	cyclohexylamine, N-methylcyclohexylamine, cycloheptanamine; it recognizes also other cyclic amines, but not simple aliphatic and aromatic amides.
protein-lysine 6-oxidase	1.4.3.13	cadaverine, benzylamine, protein 5-hydroxylysine; it catalyzes collagen and elastin cross-linking
polyamine oxidase (propane-1,3-diamine-forming)	1.5.3.14	spermidine, less efficient for N1-acetylspermine and spermine
N8-acetylspermidine oxidase (propane-1,3-diamine-forming)	1.5.3.15	N8-acetylspermine, N1-acetylspermine
spermine oxidase	1.5.3.16	spermine, norspermine, N1-acetylspermine
non-specific polyamine oxidase (former polyamine oxidase)	1.5.3.17	spermine, spermidine, acetylspermidine, thermospermine; different properties depending on the source organism
catechol oxidase (polyphenol oxidase)	1.10.3.1	(epi)catechin, catechol, dopamine, epigallocatechin, 4-methylcatechol, caffeic acid, gallic acid, quercetin, pyrogallol
laccase (polyphenol oxidase A)	1.10.3.2	catechol, l-DOPA, melanin, naphthol, ABTS (chromogenic), dichlorophenol, 2-methylphenol, 4-methylcatechol, caffeic acid, DOPA, ferulic acid, phenol, vanillic acid, 4-aminophenol, *o/p*-phenylenediamine
tyrosinase (monophenol, polyphenol oxidase; polyphenol oxidase B)	1.14.18.1	phenol, catechol, chlorophenol, dl-tyrosine, dl-DOPA, caffeic acid, gallic acid, chlorogenic acid, (epi)catechin, pyrogallol, luteolin, *p*-coumaric acid

## Data Availability

No new data were created or analyzed in this study. Data sharing is not applicable to this article.
